# Tomoregulin-1 prevents cardiac hypertrophy after pressure overload in mice by inhibiting TAK1-JNK pathways

**DOI:** 10.1242/dmm.021303

**Published:** 2015-08-01

**Authors:** Dan Bao, Dan Lu, Ning Liu, Wei Dong, Ying-Dong Lu, Chuan Qin, Lian-Feng Zhang

**Affiliations:** 1 Key Laboratory of Human Disease Comparative Medicine, Ministry of Health, Institute of Laboratory Animal Science, Chinese Academy of Medical Sciences & Comparative Medical Center, Peking Union Medical College, Beijing 100021, China; 2 Key Laboratory of Human Disease Animal Model, State Administration of Traditional Chinese Medicine, Institute of Laboratory Animal Science, Chinese Academy of Medical Sciences & Comparative Medical Center, Peking Union Medical College, Beijing 100021, China

**Keywords:** Tomoregulin-1, Transgenic mice, Cardiac hypertrophy, Pressure overload

## Abstract

Cardiac hypertrophy is associated with many forms of heart disease, and identifying important modifier genes involved in the pathogenesis of cardiac hypertrophy could lead to the development of new therapeutic strategies. Tomoregulin-1 is a growth factor that is primarily involved in embryonic development and adult central nervous system (CNS) function, and it is expressed abnormally in a variety of CNS pathologies. Tomoregulin-1 is also expressed in the myocardium. However, the effects of tomoregulin-1 on the heart, particularly on cardiac hypertrophy, remains unknown. The aim of the study is to examine whether and by what mechanism tomoregulin-1 regulates the development of cardiac hypertrophy induced by pressure overload. In this study, we found that tomoregulin-1 was significantly upregulated in two cardiac hypertrophy models: cTnT^R92Q^ transgenic mice and thoracic aorta constriction (TAC)-induced cardiac hypertrophy mice. The transgenic overexpression of tomoregulin-1 increased the survival rate, improved the cardiac geometry and functional parameters of echocardiography, and decreased the degree of cardiac hypertrophy of the TAC mice, whereas knockdown of tomoregulin-1 expression resulted in an opposite phenotype and exacerbated phenotypes of cardiac hypertrophy induced by TAC. A possible mechanism by which tomoregulin-1 regulates the development of cardiac hypertrophy in TAC-induced cardiac hypertrophy is through inhibiting TGFβ non-canonical (TAK1-JNK) pathways in the myocardium. Tomoregulin-1 plays a protective role in the modulation of adverse cardiac remodeling from pressure overload in mice. Tomoregulin-1 could be a therapeutic target to control the development of cardiac hypertrophy.

## Abstract (precis)

**Summary:** Tomoregulin-1 is primarily involved in embryonic development and adult CNS function. To the authors' knowledge, this is the first study about the function of tomoregulin-1 on cardiac hypertrophy in mice.

## INTRODUCTION

Tomoregulin-1, also named TMEFF1 or TR1, was first identified in *Xenopus laevis* as X7365, and its ortholog has been detected during mouse development and in humans, where it maps to human chromosome 9q31 (Eib et al., 1998; Morais da Silva et al., 2001). It is a type Ι transmembrane protein that contains one epidermal growth factor (EGF)-like motif and two follistatin (FS) domains in its extracellular region, and a short conserved cytoplasmic tail following the transmembrane region (Eib and Martens, 1996). Precursors of tomoregulin-1 are glycosylated proteins, and cleavage results in release of the ectodomain as a soluble protein. Soluble tomoregulin-1 stimulates phosphorylation of erbB-4 tyrosine, a receptor of the EGF/erbB family (Lin et al., 2003; Uchida et al., 1999).

It has been shown that tomoregulin-1 is predominantly expressed in embryonic development and in the central nervous system (CNS) of mice (Eib et al., 2000), involving both normal development in the embryo and the maintenance of normal CNS function in adults. Tomoregulin-1 selectively inhibits nodal in *Xenopus* ectodermal explants through its FS and EGF domains, and it also inhibits mesodermal, but not epidermal, induction by the bone morphogenetic proteins (BMPs) through its cytoplasmic tail. Both nodal and BMPs are two main classes of the transforming growth factor beta (TGFβ) ligands, which play different roles in the patterning of early embryos (Chang et al., 2003). Furthermore, tomoregulin-1 is abnormally expressed in a variety of CNS pathologies. The majority of brain tumors have lower levels of tomoregulin-1 expression than does normal brain tissue, and the ectopic expression of tomoregulin-1 results in the inhibition of cancer cell growth (Gery et al., 2003). In the 6-hydroxy-dopamine-induced cell-culture model of Parkinson's disease, the expression of tomoregulin-1 is also significantly decreased (Noelker et al., 2012). However, tomoregulin-1 is upregulated in a feline model of GM2 gangliosidosis, which is an authentic biochemical and morphological replica of human Sandhoff disease (Cork et al., 1977), and tomoregulin-1 shows a shift in its intracellular location to the nucleus and seems to function in primary dendrite initiation (Siegel et al., 2002).

Tomoregulin-1 is also expressed in heart tissue (Kanemoto et al., 2001) and can directly bind to the nodal co-receptor Cripto (Harms and Chang, 2003), which is expressed in the myocardium of the developing heart and is involved in cardiac development (Dono et al., 1993; Schnee and Hsueh, 2000; Xu et al., 1998, 1999). Collectively, these data suggest that tomoregulin-1 could be involved in the pathogenesis of heart disease. However, the effect of tomoregulin-1 on the heart, particularly on cardiac hypertrophy, remains unknown. In the present study, we found that the expression of tomoregulin-1 is increased in the myocardium of two mouse models – the cTnT^R92Q^ cardiac hypertrophy transgenic mice and the thoracic aorta constriction (TAC)-induced cardiac hypertrophy mice – and we proposed a possible mechanism by which tomoregulin-1 regulates the development of cardiac hypertrophy in myocardium-specific tomoregulin-1-knockdown and myocardium-specific tomoregulin-1-overexpression mice.

## RESULTS

### Expression of tomoregulin-1 in wild-type and cardiac hypertrophy mouse myocardium

**Fig. 1.**
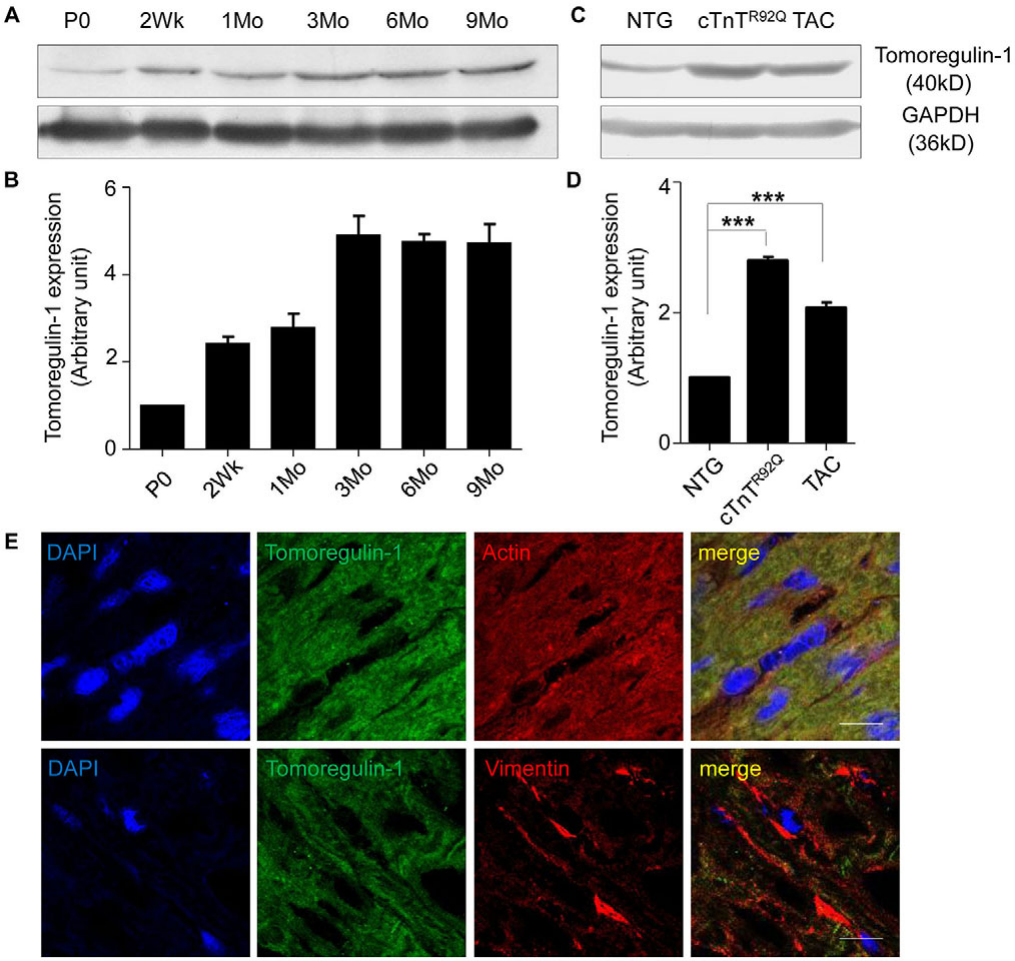
**Expression of tomoregulin-1 in wild-type and cardiac hypertrophy mouse myocardium.** Protein level of tomoregulin-1 in the myocardium from wild-type (WT) mice at postnatal day 0, 2 weeks and 1, 3, 6 and 9 months of age (A,B) and in the myocardium of mouse models (C,D) – cTnT^R92Q^ transgenic mice at 3 months of age and thoracic aorta constriction (TAC) mice at 4 weeks after TAC surgery – were detected by western blotting, using GAPDH as normalization. NTG, non-transgenic. (B,D) Bars represent the relative levels quantified by densitometry using ImageJ software (*n*=3 independent experiments, ***P<0.001 versus WT mice). (E) Immunofluorescence staining for tomoregulin-1 (green) in the myocardium of WT mice. Blue, DAPI (nucleic acid); red, α skeletal muscle sarcomeric actin (myocardial cells) or Vimentin (cardiac fibroblasts). Scale bars: 50 μm.

We assessed the expression of tomoregulin-1 in the myocardium from wild-type mice (WT) of different ages (Fig. 1A,B). Its expression was low in the neonatal myocardium and increased in the adult myocardium. We detected the expression of tomoregulin-1 in the cardiac hypertrophy myocardium from cTnT^R92Q^ mice at 3 months of age and TAC-treated mice at 4 weeks after TAC operation, and its expression was approximately 2.8- and 2.1-fold of the WT mice, respectively (Fig. 1C,D, *n*=3 independent experiments, *P*<0.001). The immunofluorescence showed that tomoregulin-1 was primarily expressed in cardiomyocytes, but not in cardiac fibroblasts (Fig. 1E).

**TRANSLATIONAL IMPACT**

**Clinical issue**

Cardiac hypertrophy occurs in many heart diseases, such as essential hypertension, myocardial infarction and valvular heart diseases. Cardiac hypertrophy is an adaptive reaction in response to increased pressure overload. Sustained pressure overload usually induces an initial compensatory hypertrophy, which can progress to pathologic cardiac hypertrophy and finally to congestive heart failure. Currently, the molecular mechanisms contributing to cardiac hypertrophy in response to pressure overload are incompletely understood. Identifying important modifier genes involved in the pathogenesis of cardiac hypertrophy might lead to the development of new therapeutic strategies.

****Results****

In this study, the authors found that the expression of tomoregulin-1 (a growth factor that is involved in embryonic development and is expressed in the brain and myocardium) was significantly increased in two cardiac hypertrophy models: cTnT^R92Q^ transgenic mice and thoracic aorta constriction (TAC)-treated mice. Transgenic overexpression of tomoregulin-1 in mice exerted a protective effect on cardiac hypertrophy and also slowed the progression from cardiac hypertrophy to heart failure induced by the TAC operation. By contrast, tomoregulin-1 knockdown exacerbated TAC-induced cardiac hypertrophy phenotypes. Moreover, TGFβ non-canonical (TAK1-JNK) signaling was one of the pathways involved in the regulatory effect of tomoregulin-1 on cardiac hypertrophy.

****Implications and future directions****

This is the first study that investigates the function of tomoregulin-1 in the heart, particularly on cardiac hypertrophy. The current results suggest that tomoregulin-1 has a protective role against cardiac hypertrophy and might represent a potential target for therapeutic strategy to control its development.

### Generation of myocardium-specific tomoregulin-1-knockdown and -overexpression mice

**Fig. 2.**
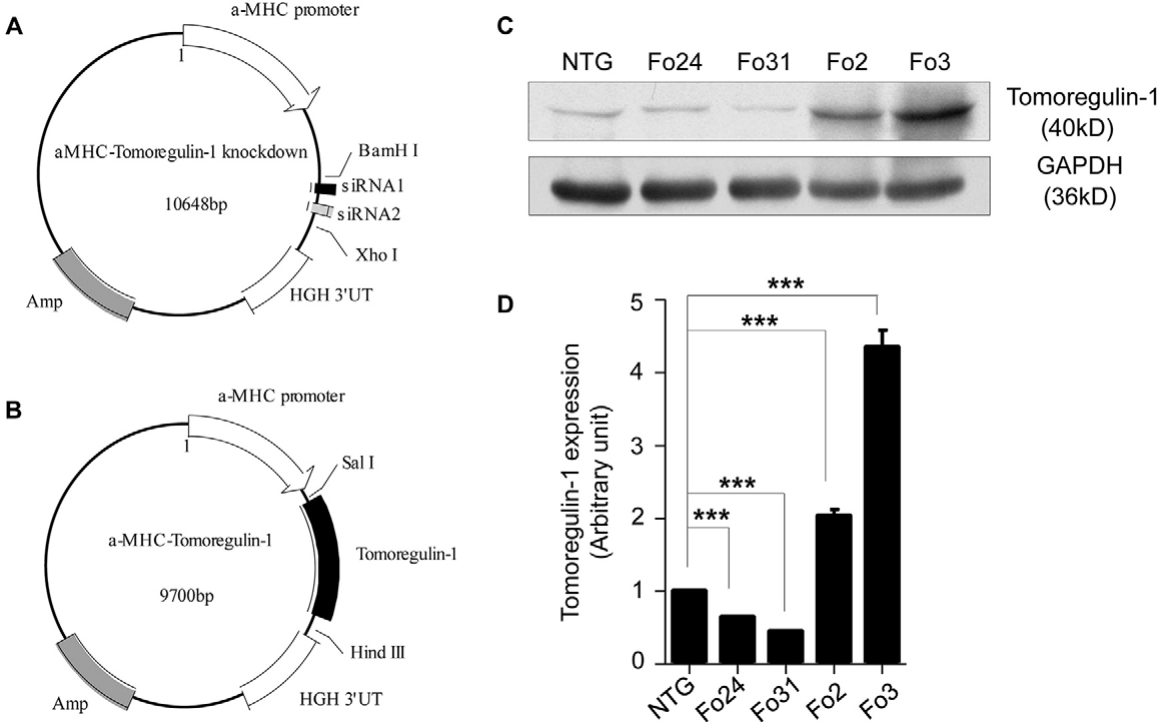
**Generation of myocardium-specific tomoregulin-1-knockdown and -overexpression mice.** The α-MHC–tomoregulin-1-knockdown construct (A) and the α-MHC–tomoregulin-1-overexpression construct (B) were generated with two siRNAs with high efficiency on knockdown of tomoregulin-1 and the full-length mouse tomoregulin-1 cDNA under the control of the α-MHC myocardium-specific promoter, respectively, and the mice were created following microinjection. The expression of tomoregulin-1 in the myocardium of two lines of tomoregulin-1-knockdown mice and two lines of tomoregulin-1-overexpression mice at 2 months of age were measured by western blot (C). The quantitative analysis of the expression of tomoregulin-1 (D) use GAPDH as normalization (*n=*3 independent experiments, ***P<0.001 versus NTG mice). NTG, non-transgenic; Fo, founder.

To determine whether tomoregulin-1 affects the cardiac geometry and function in TAC-induced cardiac hypertrophy myocardium, we generated myocardium-specific tomoregulin-1-knockdown mice (Fig. 2A) and tomoregulin-1-overexpression mice (Fig. 2B). The expression of tomoregulin-1 was reduced by approximately 35% and 55%, respectively, in two knockdown mouse lines (founder 24 and 31), and the expression of tomoregulin-1 was approximately two- and four-fold of the non-transgenic (NTG) mice, respectively, in two overexpression mouse lines (founder 2 and 3) (Fig. 2C,D). The two knockdown mouse lines showed similar phenotypes, including thin-walled ventricles and larger left ventricular diameters on M-mode echocardiography, and the two overexpression mouse lines showed similar phenotypes, including thick-walled ventricles and smaller left ventricular on M-mode echocardiography. Therefore, we used the founder 31 of knockdown mice and the founder 3 of overexpression mice to study the possible effect of tomoregulin-1 on the development of cardiac hypertrophy. The data of another line for tomoregulin-1-knockdown and tomoregulin-1-overexpression mice are shown in supplementary material Tables S1-S5.

### Echocardiography analysis of myocardium-specific tomoregulin-1-knockdown and -overexpression mice in baseline

**Fig. 3.**
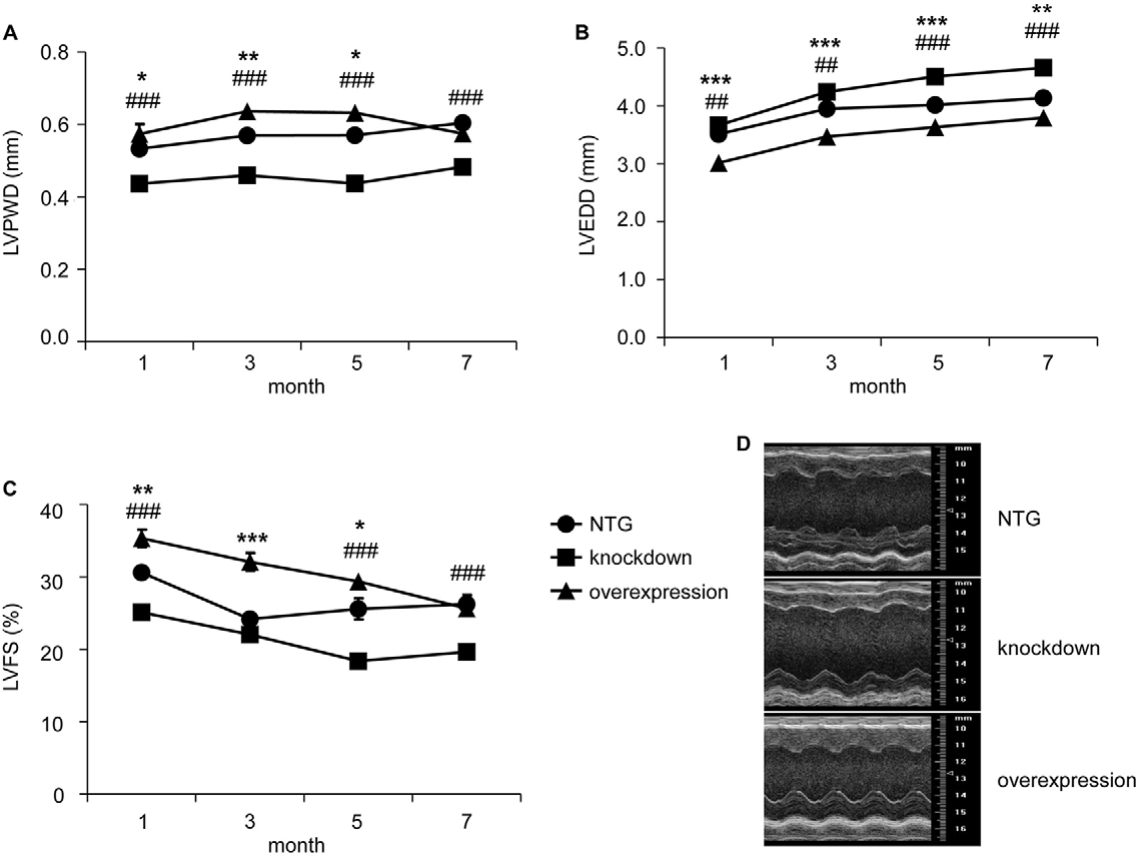
**Echocardiography analysis of myocardium-specific tomoregulin-1-knockdown and -overexpression mice in baseline.** Echocardiographic parameters of left ventricular (LV) posterior wall at end-diastole (LVPWD) (A), LV diameter at end-diastole (LVEDD) (B) and LV fractional shortening (LVFS) (C) were analyzed in non-transgenic (NTG) (*n*=65, 55, 36 and 27), tomoregulin-1-knockdown (*n*=41, 33, 25 and 15) and tomoregulin-1-overexpression (*n*=23, 41, 38 and 40) mice at 1, 3, 5 and 7 months of age (*P<0.05, **P<0.01, ***P<0.001 overexpression versus NTG mice, ^##^*P*<0.01, ^###^*P*<0.001 knockdown versus NTG mice). The M-mode echocardiomyopathy figures of NTG, tomoregulin-1-knockdown and tomoregulin-1 overexpression mice at 5 months of age (D) are shown.

We analyzed the cardiac geometry and function in myocardium-specific tomoregulin-1-knockdown and tomoregulin-1-overexpression mice using echocardiography and verified the effect of tomoregulin-1 on the heart (Fig. 3, supplementary material Tables S1-S4 and Fig. S1).

The tomoregulin-1-knockdown mice presented thin-walled ventricles and larger left-ventricular diameters compared with the NTG mice [Fig. 3A,B, left ventricular posterior wall thickness at end-diastole (LVPWD) decreased 17.7%, 19.0%, 23.4% and 20.0%, respectively, at 1, 3, 5 and 7 months of age; left ventricular diameter at end-diastole (LVEDD) increased 4.4%, 7.4%, 12.2% and 12.5%, respectively, at 1, 3, 5 and 7 months of age (*n*=41, 33, 25 and 15, *P*<0.05)]. The tomoregulin-1-overexpression mice showed thick-walled ventricles and smaller left ventricular diameters before 5 months of age [Fig. 3A,B; LVPWD increased 8.0%, 12.2% and 10.9%, respectively, at 1, 3 and 5 months of age; LVEDD decreased 14.1%, 12.1% and 9.6%, respectively, at 1, 3 and 5 months of age (*n*=23, 41, 38 and 40, *P*<0.05)]. The thick-walled ventricle phenotype caused by tomoregulin-1 overexpression was not obvious after 5 months of age compared with that of the NTG mice. Furthermore, cardiac function was also affected by tomoregulin-1 knockdown, indicated by decreased left ventricular percent fractional shortening (LVFS) compared with that of the NTG mice [Fig. 3C, LVFS decreased 16.3%, 9.3%, 28.2% and 25.2%, respectively, at 1, 3, 5 and 7 months of age (*n*=41, 33, 25 and 15, *P*<0.05)]. The cardiac function was improved by tomoregulin-1 overexpression, indicating increased LVFS compared with that of the NTG mice [Fig. 3C, LVFS increased 14.4%, 25.1% and 13.7%, respectively, at 1, 3 and 5 months of age (*n*=23, 41 and 38, *P*<0.05)].

### Tomoregulin-1 increases the survival rate and improves cardiac morphology and function after pressure overload

**Fig. 4.**
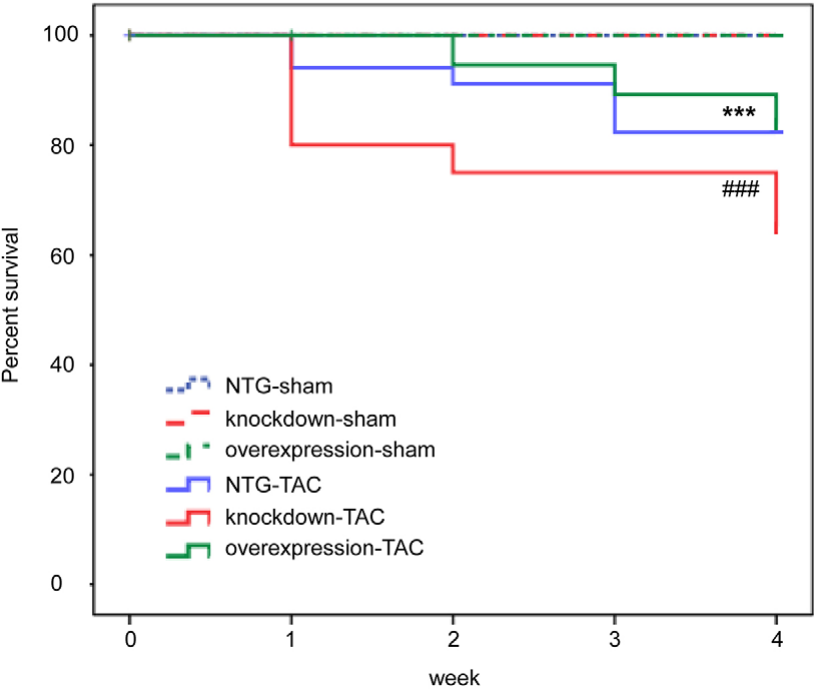
**Tomoregulin-1 increases the survival rate after pressure overload.** Cumulative mouse mortality data of the non-transgenic (NTG; *n*=55 and 35), tomoregulin-1-knockdown (*n*=33 and 21) and tomoregulin-1-overexpression (*n*=41 and 39) mice were recorded between 1 to 4 weeks after sham and TAC operation (***P<0.001 overexpression with TAC operation versus NTG mice with TAC operation, ^###^*P*<0.001 knockdown with TAC operation versus NTG mice with TAC operation).

The cumulative mouse mortality data of the NTG, tomoregulin-1-knockdown and tomoregulin-1-overexpression mice were recorded between 1 to 4 weeks after the TAC operation. The survival rate was all 100% in the NTG (*n*=55), tomoregulin-1-knockdown (*n*=33) and tomoregulin-1-overexpression mice (*n*=41) of the sham group. The survival rate of the tomoregulin-1-knockdown mice with TAC (*n*=21) was significantly reduced by 13.3%, 15.2%, 6.7% and 1.9%, respectively, at 1, 2, 3 and 4 weeks after the TAC operation (*P*<0.001) compared to the NTG mice with TAC (*n*=35), whereas the survival rate of the tomoregulin-1-overexpression mice with TAC (*n*=39) was significantly increased by 5.7%, 3.4%, 6.9% and 16.0%, respectively, at 1, 2, 3 and 4 weeks after the TAC operation (*P*<0.001) compared to the NTG mice with TAC (*n*=35) (Fig. 4).

**Fig. 5.**
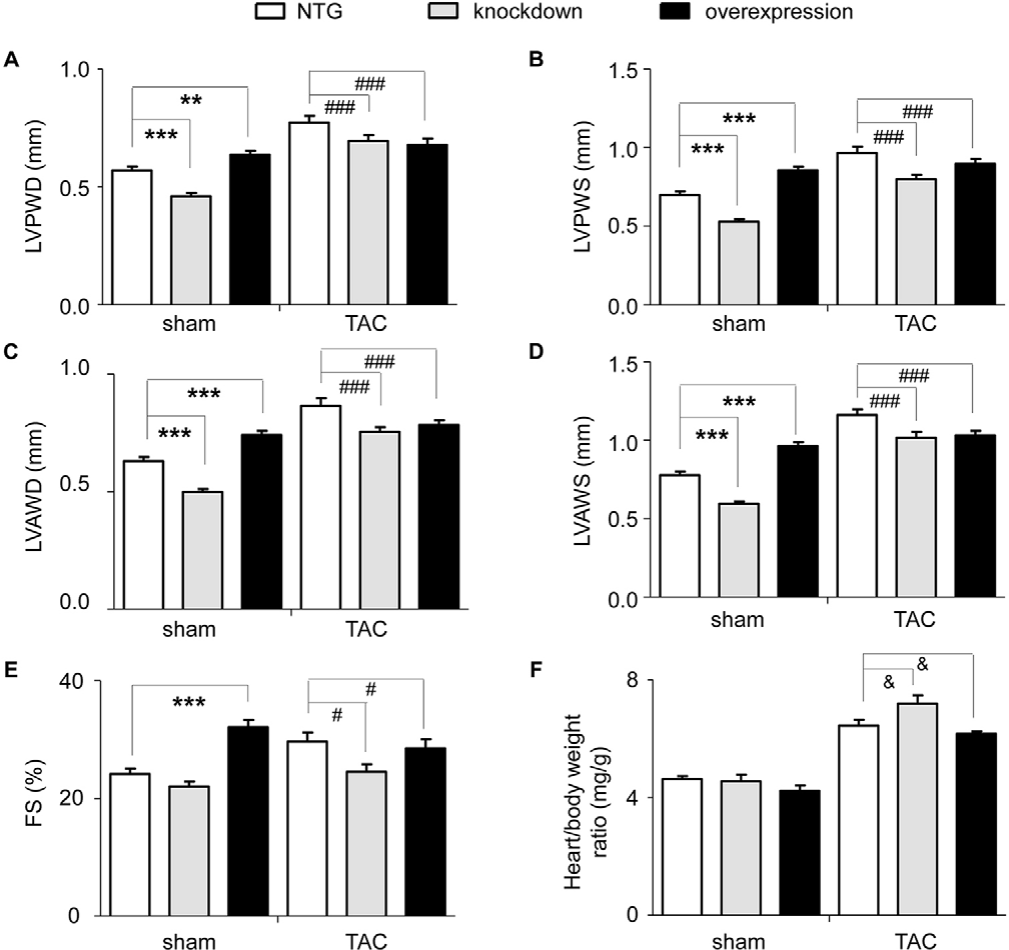
**Tomoregulin-1 improves cardiac morphology and function after pressure overload.** Echocardiographic parameters of LVPWD (A), LV posterior wall at end-systole (LVPWS) (B), LV anterior wall at end-diastole (LVAWD) (C), LV anterior wall at end-systole (LVAWS) (D) and left ventricular percent fractional shortening (LVFS) (E) were analyzed in non-transgenic (NTG; *n*=55 and 28), tomoregulin-1-knockdown (*n*=33 and 14) and tomoregulin-1-overexpression (*n*=41 and 28) mice at 4 weeks after the sham and TAC operation (**P<0.01, ***P<0.001 versus NTG mice in sham group, ^#^*P*<0.05, ^###^*P*<0.001 versus the percent change of NTG mice). (F) Heart-weight:body-weight ratio was calculated in NTG (*n*=18 and 12), tomoregulin-1-knockdown (*n*=10 and 8) and tomoregulin-1-overexpression (*n*=16 and 10) mice at 4 weeks after sham and TAC operation (^&^*P*<0.05 versus NTG mice).

Furthermore, the LVPWD, left ventricular posterior wall thickness at end-systole (LVPWS), left ventricular anterior wall thickness at end-diastole (LVAWD), left ventricular anterior wall thickness at end-systole (LVAWS) and LVFS of all surviving mice were detected using echocardiography at 4 weeks after the sham and TAC operation for NTG, tomoregulin-1-knockdown and tomoregulin-1-overexpression mice (Fig. 5A-E and supplementary material Tables S5, S6). Because there are significant differences in the echocardiographic parameters of the sham group, the percent change of the cardiac echocardiography parameters represents the development of cardiac hypertrophy induced by TAC. The percent change of LVPWD, LVPWS, LVAWD and LVAWS were 36.4%, 39.6%, 38.5% and 50.8% in NTG mice, 51.1%, 51.0%, 51.3% and 70.5% in knockdown mice and 6.6%, 4.8%, 5.7% and 7.2% in overexpression mice, respectively. The results indicated that tomoregulin-1 knockdown significantly increased the cardiac geometrical changes of cardiac hypertrophy induced by TAC; however, tomoregulin-1 overexpression significantly reduced the geometrical changes induced by TAC. Following the appearance of the cardiac hypertrophy induced by TAC, the LVFS was also increased to compensate for the heart dysfunction. However, the LVFS was not increased in overexpression mice. The percent change of LVFS was 22.1% in NTG mice, 11.3% in knockdown mice and −11.1% in overexpression mice (*P*<0.05). The mice were sacrificed after 4 weeks of TAC, and the hearts were weighed and the heart-weight:body-weight ratio was calculated (Fig. 5F). The tomoregulin-1-knockdown mice showed a significantly increased heart-weight:body-weight ratio (*n*=8, *P*<0.05), and the tomoregulin-1-overexpression mice had a significantly decreased the heart-weight:body-weight ratio compared with that of the NTG mice (*n*=10, *P*<0.05). The parameters of the M-mode echocardiography at 16 weeks after the TAC operation also suggested that the TAC-induced progression from cardiac hypertrophy to heart failure was inhibited by the overexpression of tomoregulin-1 (data not shown, supplementary material Table S7).

### Tomoregulin-1 improves cardiac pathological changes after pressure overload

**Fig. 6.**
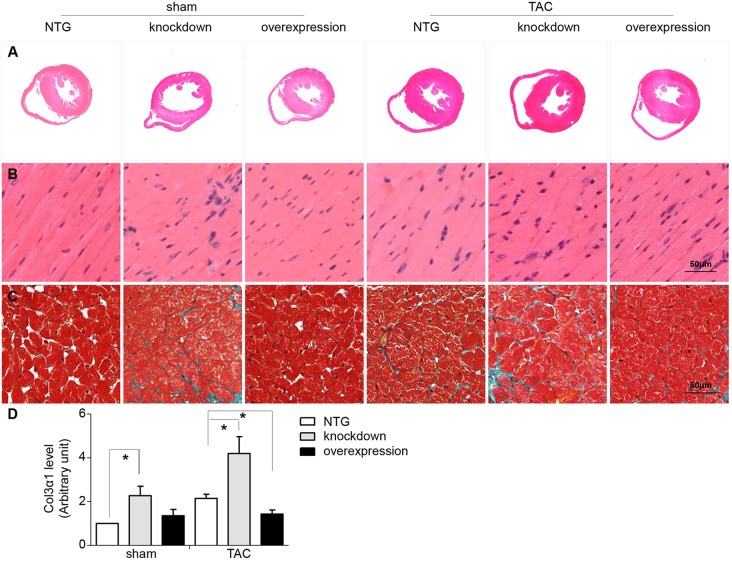
**Tomoregulin-1 improves cardiac pathological changes after pressure overload.** H&E staining patterns of the whole-heart cross-sections from mice at 4 weeks after sham and TAC operation (A). Magnification of H&E-stained sections of the left ventricle (LV), showing disparate pathological changes (B). Magnification of Masson-trichrome-stained sections of LV; myocytes, stained red; collagenous tissue, stained green (C). The expression of Col3α1 was detected by real-time PCR, and the quantitative analysis of the expression of Col3α1 made using GAPDH for normalization (D; *n=*3 independent experiments, *P<0.05 versus NTG mice). NTG, non-transgenic.

The mice were sacrificed after 4 weeks of the sham and TAC operation, and the morphological changes of the myocardium were observed under light microscopy. Myocardial disarray (Fig. 6B) and fibrosis (Fig. 6C) were clearly observed in the myocardium from the tomoregulin-1-knockdown mice. However, the pathological changes of TAC operation were significantly inhibited by tomoregulin-1 overexpression. Moreover, the expression of Col3α1, which is responsible for collagen deposition, was significantly increased in tomoregulin-1-knockdown mice in the sham and TAC group, compared with NTG and overexpression mice (Fig. 6D, *P*<0.05). However, the pathological increase of the Col3α1 in the TAC group was significantly inhibited by tomoregulin-1 overexpression (*P*<0.05).

### Tomoregulin-1 inhibits the TGFβ non-canonical (TAK1-JNK) pathways after pressure overload

**Fig. 7.**
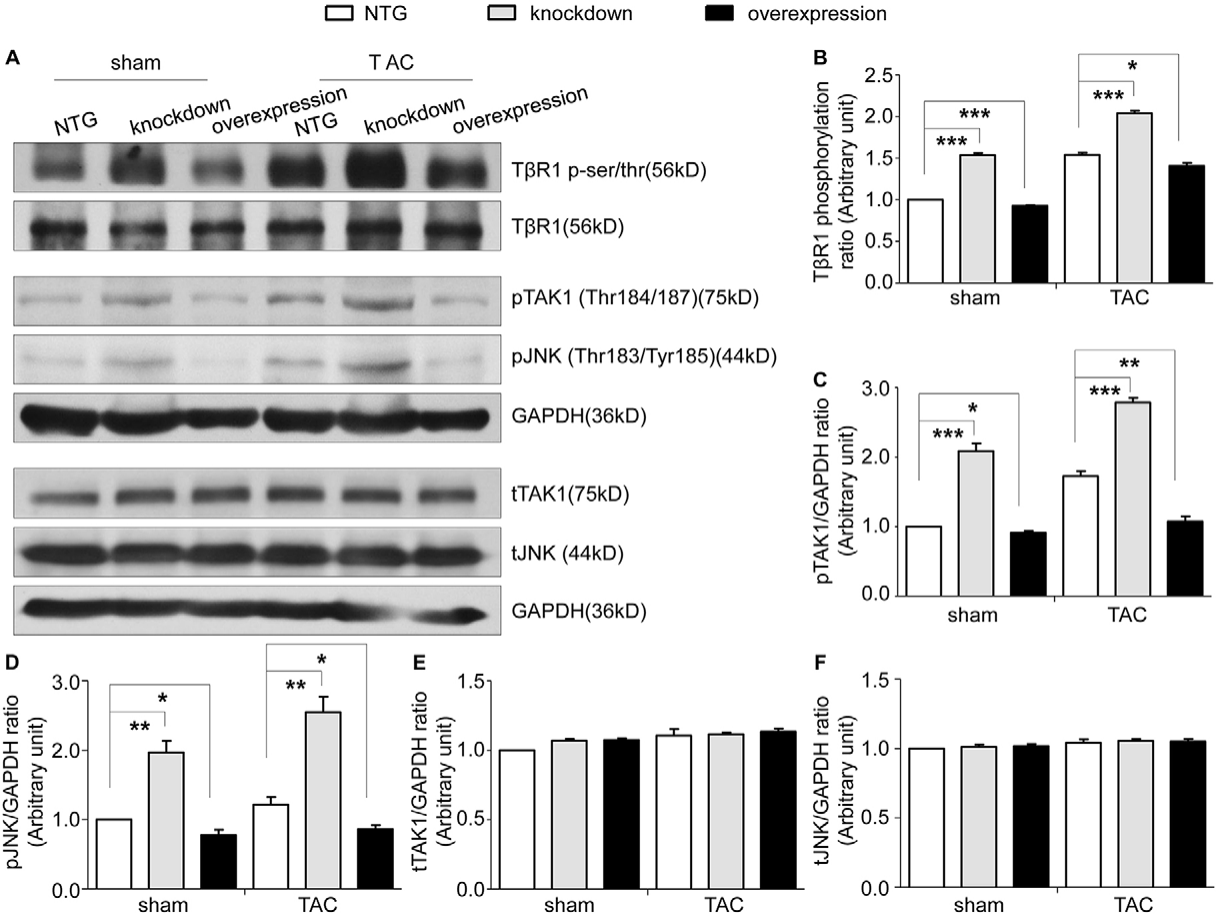
**Tomoregulin-1 inhibits the TGFβ non-canonical (TAK1-JNK) pathways after pressure overload.** The phosphorylation level of TβR1, TAK1 and JNK were measured by western blot in the myocardium of non-transgenic (NTG), tomoregulin-1-knockdown and tomoregulin-1-overexpression mice at 4 weeks after sham and TAC operation (A). The quantitative analysis of the level of phosphorylated TβR1 (B) use TβR1 for normalization, phosphorylated TAK1 (C) and JNK (D), and total TAK1 (E) and JNK (F), use GAPDH for normalization (*n=*3 independent experiments, *P<0.05, **P<0.01, ***P<0.001 versus NTG mice).

Tomoregulin-1 is located in cardiomyocytes and contains two FS domains in its extracellular region, which interact with TGFβs. TGFβ signaling suppression in cardiomyocytes is required to protect against the murine pathological response to sustained pressure overload, which involves non-canonical (TAK1-JNK) signaling. Therefore, we measured the phosphorylation level of the TGFβ type 2 receptor (TβR2), TGFβ type 1 receptor (TβR1), TGFβ-activated kinase 1 (TAK1) and c-Jun N-terminal kinase (JNK) in the myocardium from the NTG, tomoregulin-1-knockdown and tomoregulin-1-overexpression mice. We found that the phosphorylation of TβR2 was comparable between the NTG and tomoregulin-1-overexpression mice (data not shown, supplementary material Fig. S2), the phosphorylation of TβR1 (Fig. 7A,B, *P*<0.05) was higher in the tomoregulin-1-knockdown mice compared with that of the NTG mice, and the phosphorylation of TAK1 and JNK (Fig. 7A,C,D, *P*<0.05) were subsequently activated, whereas the tomoregulin-1-overexpression mice had the lowest level of phosphorylation of TβR1, TAK1 and JNK after pressure overload, suggesting that overexpression of tomoregulin-1 significantly inhibited the activation of the TGFβ non-canonical (TAK1-JNK) signaling pathways in TAC-induced cardiac hypertrophy.

## DISCUSSION

In the present study, we demonstrated that the expression of tomoregulin-1 was significantly increased in the myocardium from two cardiac hypertrophy models: cTnT^R92Q^ transgenic mice and TAC mice (Fig. 1). The transgenic overexpression of tomoregulin-1 increased the survival rate (Fig. 4), improved the cardiac geometry and functional parameters of echocardiography (Fig. 5), decreased the degree of cardiac hypertrophy (Fig. 5), and reduced collagen accumulation in the interstitial space of the TAC mice (Fig. 6). We also found that TGFβ non-canonical (TAK1-JNK) signaling was involved in the effect of tomoregulin-1 in the cardiac hypertrophy of mice (Fig. 7).

According to the previous study, tomoregulin-1 was considered a member of the EGF superfamily of peptides (Saloman et al., 2000) because it contained one EGF-like motif in its extracellular region which was highly conserved compared to EGF/NRG family growth factors with the exception of an arginine-to-histidine substitution at position 39 (Arg→His 39), which drastically reduced the affinity for the EGF receptor, erbB-4, because this region is essential for EGF receptor recognition (Engler et al., 1990; Hommel et al., 1991). Soluble tomoregulin-1 stimulates erbB-4 tyrosine phosphorylation in MKN28 gastric cancer cells, suggesting that tomoregulin-1 might be a ligand for the erbB-4 or erbB-4-related receptor tyrosine kinase (Uchida et al., 1999). Therefore, we hypothesized that tomoregulin-1 would affect the phosphorylation level of erbB-4. However, we found that the phosphorylation level of erbB-4 was comparable among the tomoregulin-1-knockdown mice, tomoregulin-1-overexpression mice and their NTG littermates, suggesting that tomoregulin-1/erbB-4 was not involved in the myocardium, at least in the current study (data not shown, supplementary material Fig. S3).

Moreover, tomoregulin-1 also contains two FS domains in its extracellular region, which interact with TGFβs (Eib and Martens, 1996). Therefore, we focused on TGFβ signaling involved in the effect of tomoregulin-1 on TAC-induced cardiac hypertrophy. TGFβ expression is increased in the myocardium in cardiac hypertrophy experimental models and human cardiac hypertrophy patients (Dobaczewski et al., 2011; Hein et al., 2003; Li et al., 1997; Rosenkranz, 2004; Villar et al., 2009), and it promotes the hypertrophic growth of cardiomyocytes (Schultz et al., 2002), fibroblast proliferation and transdifferentiation into myofibroblasts (Petrov et al., 2002), which collectively contribute to the progression of cardiac hypertrophy and fibrosis (Villar et al., 2013). We found that the expression of tomoregulin-1 was also increased significantly in the myocardium from the cTnT^R92Q^ transgenic mice and TAC-induced cardiac hypertrophy mice (Fig. 1), and we proposed that the coordinated myocardial expression pattern of tomoregulin-1 with TGFβs inhibited the hypertrophic and fibrogenic effects of this potent cytokine. Our results in the tomoregulin-1-knockdown and tomoregulin-1-overexpression mice subjected to TAC further supported this hypothesis. The overexpression of tomoregulin-1 significantly reduced the increase of LVPWD, LVPWS, LVAWS and LVAWD (Fig. 5), decreased the degree of cardiac hypertrophy (Fig. 5), and reduced collagen accumulation in the interstitial space (Fig. 6), which collectively contribute to the slowed progression of cardiac hypertrophy, whereas knockdown of tomoregulin-1 expression exacerbated phenotypes of cardiac hypertrophy induced by TAC. We then found that the phosphorylation level of TβR1 was inhibited in tomoregulin-1-overexpression mice, as well as the phosphorylation level of TAK1 and its downstream kinase JNK compared with NTG mice, whereas the phosphorylation level of TβR1, TAK1 and JNK were significantly activated in tomoregulin-1-knockdown mice. TAK1 is a component of TGFβ non-canonical (Smad-independent) signaling involving direct kinase activation (Derynck and Zhang, 2003) and seems to be important in heart disease (Koitabashi et al., 2011; Zhang et al., 2000). TAK1 is activated in cardiomyocytes following pressure overload generated by TAC, and cardiac-specific overexpression of activated TAK1 in a transgenic mouse leads to cardiac hypertrophy and heart failure. Furthermore, a dominant-negative TAK1 protein can inhibit TGFβ-induced hypertrophic events in cardiomyocytes (Ono et al., 2003; Zhang et al., 2000). JNK is a TAK1 downstream kinase and is also activated in *in vivo* models of pressure overload hypertrophy (Fischer et al., 2001; Sopontammarak et al., 2005), and adenoviral-mediated overexpression of the dominant-negative mutant of the immediate upstream activator of JNK blocks JNK activation and inhibits pressure-overload cardiac hypertrophy (Choukroun et al., 1999). Collectively, these data suggest that the TGFβ non-canonical (TAK1-JNK) pathway is an important regulator of the hypertrophic response and is involved in the effect of tomoregulin-1 on the cardiac hypertrophy of mice.

In conclusion, we found that tomoregulin-1 plays a protective role in the maladaptive hypertrophy caused by pressure-overload stress, and TGFβ non-canonical (TAK1-JNK) signaling is one of the pathways involved in the regulatory effect of tomoregulin-1 on cardiac hypertrophy.

## MATERIALS AND METHODS

### Animals

Five siRNAs against the tomoregulin-1 gene were designed using Invitrogen's RNAi Designer (www.invitrogen.com/rnai) and were inserted into the pcDNA™6.2-GW/EmGFP-miR vector (Invitrogen, BLOCK-iT™ Pol II miR RNAi Expression Vector Kit). The knockdown effects on tomoregulin-1 were screened in 293T cells by co-transfection of the siRNA and the mouse tomoregulin-1 cDNA. Two siRNAs with high efficiency showed synergic effects on the knockdown of tomoregulin-1 in 293T cells (supplementary material Fig. S4) and were moved from pcDNA™6.2-GW/EmGFP-miR vector into a myocardium-specific expression vector under a α-myosin heavy chain (α-MHC) promoter, which was constructed using the α-MHC promoter and PBR322 backbone and used commonly for myocardium-specific gene expression *in vivo* (Sano et al., 2002; Sidhu et al., 2005). The sequences for siRNA1 and siRNA2 were 5′-TGCTGACTAAAGTCCGTCTTCTCACAGTTTTGGCCACTGACTGACTGTGAGAACGGACTTTAGT-3′ (siRNA1) and 5′-TGCTGTATAATGGCGATCTGTACTGCGTTTTGGCCACTGACTGACGCAGTACATCGCCATTATA-3′ (siRNA2). The myocardium-specific tomoregulin-1-knockdown mouse was generated by microinjection and was genotyped by PCR with the primers 5′-CGACAACCACTACCTGAGCAC-3′ and 5′-GCCCTCTAGATCAACCACTTTG-3′. For genotyping, a 525-bp fragment of the transgenic gene was amplified with 30 PCR cycles consisting of 94°C for 30 s, 60°C for 30 s and 72°C for 40 s.

The full-length mouse tomoregulin-1 cDNA was cloned into an expression plasmid under the α-MHC promoter. The myocardium-specific tomoregulin-1-overexpression mouse was generated by microinjection and was genotyped by PCR with the primers 5′-GCCAGTTTCAGTGCCATACA-3′ and 5′-GTTCTCCGCATCCTCGTC-3′. For genotyping, a 263-bp fragment of the transgenic gene was amplified with 30 PCR cycles consisting of 94°C for 30 s, 56°C for 30 s and 72°C for 30 s. The expression of tomoregulin-1 was screened by western blot analysis using a goat antibody (R&D). The α-MHC-cTnT^R92Q^ (referred to as cTnT^R92Q^) cardiac hypertrophy transgenic mice were previously generated in our laboratory. R92Q is a missense mutation in the strong tropomyosin-binding region of cardiac troponin T (cTnT), and the cTnT^R92Q^ transgenic mice manifest ventricular wall hypertrophy, reduced ventricular chamber and diastolic dysfunction, and have a pathological phenotype similar to that of human hypertrophic cardiomyopathy (Thierfelder et al., 1994).

All mice (*Mus musculus*) used in this study were maintained on a C57BL/6J genetic background and were bred in an AAALAC-accredited facility. Mice were housed in a room kept at 23±2°C with 12:12 h light/dark cycle and provided with standard food and water *ad libitum*. The ratio of genders used in this study was approximately 1:1. The use of animals was approved by the Animal Care and Use Committees of The Institute of Laboratory Animal Science of Peking Union Medical College (ILAS-GC-2012-001).

### TAC-induced cardiac hypertrophy mouse model

α-MHC–tomoregulin-1-knockdown mice (referred to as knockdown), α-MHC–tomoregulin-1-overexpression mice (referred to as overexpression) and non-transgenic (NTG) littermates at 8 to 10 weeks of age were used for the TAC operation. Briefly, the mice were anesthetized by intraperitoneal injection of 18 ml/kg body weight tribromoethanol. The surgery was performed under passive respiration using a ventilator (125∼150 times/min, Kent Scientific Torrington, CT 06790, USA). The aorta was constricted between the origin of the right innominate and left common carotid arteries with a 6/0 polypropylene ligature using a blunted 27-gauge (0.41-mm OD) needle as a calibrator. The Doppler analysis was performed to detect the innominate artery (IA) and left common carotid artery (LCCA) blood flow 1 week after TAC and the LVPWD 4 weeks after TAC, and the relativity was accounted (Wu et al., 2012). The mice with the value of the IA/LCCA flow ratio from 5.9 to 10.7 were going to develop a comparable pressure gradient and were used in the present study. The sham procedure was identical except that the aorta was not ligated. Each surgical procedure was completed within 30 min to maintain the body temperature at 37°C. If dehydration occurred, intraperitoneal injection of 0.9% saline solution would be given to mice. All surviving mice were selected for follow-up echocardiography at 4 weeks after surgery.

### Echocardiography

M-mode echocardiography was performed on tomoregulin-1-knockdown and -overexpression mice as well as their littermates with the small animal echocardiography analysis system (Vevo770, Canada) as previously described (Lu et al., 2012).

### Survival analysis

The cumulative percent mortality in the sham and TAC groups of mice was calculated each week, and the data from 1 to 4 weeks after surgery were summarized. Upon the death of each mouse, the body was autopsied by a pathologist and the morphological and pathological changes of the heart were recorded. Kaplan–Meier curves for the survival analysis were compared by the log-rank test (SPSS 16.0 software).

### Histological analysis

For light microscopy, the mice were euthanized by cervical dislocation at 12 to 14 weeks of age (4 weeks after surgery) and the cardiac tissue was fixed in 4% formaldehyde and mounted in paraffin blocks. The sections were stained with hematoxylin and eosin (H&E) or Masson trichrome as previously described (Lu et al., 2012) and analyzed using the Aperio Image Scope v8.2.5 software. The myocytes were analyzed by an observer blinded to the mouse genotypes.

### RNA extraction, quantification and real-time PCR

The mice were euthanized by cervical dislocation at 12 to 14 weeks of age (4 weeks after surgery), and the total RNA was isolated from the myocardium using TRIzol Reagent (Invitrogen, USA). First-strand cDNA was synthesized from 2 µg of total RNA using random hexamer primers and Superscript III reverse transcriptase according to the manufacturer's protocol (Invitrogen, USA). Procollagen type III α1 (*Col3α1*) mRNA was detected by real-time PCR using GAPDH for normalization under standard conditions (primers: for Col3α1, forward 5′-CTCAAGAGCGGAGAATACTGG-3′ and reverse 5′-CAATGTCATAGGGTGCGATA-3′; for GAPDH, forward 5′-CAAGGTCATCCATGACAACTTTG-3′ and reverse 5′-GTCCACCACCCTGTTGCTGTAG-3′).

### Immunofluorescence staining

The heart sections were prepared using standard pathological procedures. The sections were fixed with ice-cold acetone for 15 min, washed with PBS, and then incubated with anti-tomoregulin-1 poly antibody (BIOSS, China), anti-α skeletal muscle sarcomeric actin and anti-vimentin antibody (Abcam, UK) overnight at 4°C, respectively. The sections were washed with PBS and incubated with secondary Alexa-Fluor-488-conjugated goat anti-rabbit IgG (Invitrogen, USA) or DyLight-conjugated, affinity-purified anti-mouse IgG (KPL, USA) for 30 min at 37°C. The sections were then rinsed again with PBS and mounted in ProLong Gold anti-fade regent (Invitrogen, USA). The sections were observed and analyzed using confocal laser-scanning microscopy (Leica TCSSP2, Germany).

### Protein extraction and immunoblotting

The mice were euthanized by cervical dislocation, and total protein lysates from the mouse myocardium were prepared as previously described (Lu et al., 2014). In addition, protein lysates for phospho-TGFβ type 2 receptor (TβR2) and TGFβ type 1 receptor (TβR1) were first prepared by immunoprecipitation with the primary antibody TβR2 (Cell Signaling Technology, USA), TβR1 (Abcam, USA) and Protein A/G PLUS-Agarose (Santa Cruz, USA). After SDS-PAGE and transfer of the bands to nitrocellulose (Millipore, USA), the membranes were incubated overnight with antibodies against tomoregulin-1 (R&D, USA), phospho-serine/threonine kinase (Abcam, USA), TβR2 (Cell Signaling Technology, USA), TβR1 (Abcam, USA), phospho-TAK1 (Thr184/187) (Cell Signaling Technology, USA), phospho-JNK (Thr183/Tyr185) (Cell Signaling Technology, USA), TAK1 (Cell Signaling Technology, USA) and JNK (Cell Signaling Technology, USA). After incubation with the appropriate secondary antibody for 1 h at room temperature, antibody binding was detected with an HRP-conjugated immunoglobulin G (Santa Cruz, USA) using a chemiluminescence detection system (Santa Cruz, USA). The quantitative analysis of the level of proteins were normalized to GAPDH, and the bands were quantified using the ImageJ software.

### Statistical analysis

The data were analyzed by one-way ANOVA for multiple groups followed by Tukey's post-hoc analysis. The data are expressed as the means±s.e.m. from individual experiments. The differences are considered significant at *P*<0.05.

## Supplementary Material

Supplementary Material
