# Supplementary material for: Tomoregulin-1 prevents cardiac hypertrophy after pressure overload in mice by inhibiting TAK1-JNK pathways
Source: Dis Model Mech. 2015 Aug 1;8(8):795–804. doi: 10.1242/dmm.021303 (PMC4527297; doi:10.1242/dmm.021303)
Supplement: Supplementary Material [file supp_8_8_795__index.html]

Supplementary Material 

# Tomoregulin-1 prevents cardiac hypertrophy after pressure overload in mice by inhibiting TAK1-JNK pathways

## DMM021303 Supplementary Material

- Supplementary Material
