# Supplementary material for: Tomoregulin-1 prevents cardiac hypertrophy after pressure overload in mice by inhibiting TAK1-JNK pathways
Source: Dis Model Mech. 2015 Aug 1;8(8):795–804. doi: 10.1242/dmm.021303 (PMC4527297; doi:10.1242/dmm.021303)
Supplement: Supplementary Material [file supp_021303_DMM021303supp.pdf]

Supplemental Tables

To verify whether the Tomoregulin-1 could affect the cardiac geometry and function in thoracic aorta constriction (TAC)-induced cardiac hypertrophy myocardium, we generated two lines of myocardium-specific Tomoregulin-1 knockdown mice (founder 24 and 31) and Tomoregulin-1 overexpression mice (founder 2 and 3). M-mode echocardiography was performed at 1, 3, 5 and 7 months of age on each line of Tomoregulin-1 knockdown (referred to as knockdown) and overexpression mice (referred to as overexpression) as well as non-transgenic (NTG) littermates with the small animal echocardiography analysis system (Vevo770, Canada). The two lines of knockdown mice showed similar phenotypes of thin-walled ventricles and larger left ventricular diameters on M-mode echocardiography, and the two lines of overexpression mice showed similar phenotypes of thick-walled ventricles and smaller left ventricular. The data were shown in the followed Supplemental Table S1-S4.

Table S1. Echocardiographic characteristics of mice at 1 months of age

Group	NTG	knockdown		overexpression	
		founder 24	founder 31	founder 2	founder 3
Number	n=65	n=14	n=41	n=13	n=24
LVEDD, mm	3.51±0.28	3.61±0.34	3.67±0.21**	2.94±0.38***	3.02±0.28***
LVESD, mm	2.47±0.39	2.69±0.30*	2.75±0.28***	1.87±0.45***	1.96±0.30***
LVPWD, mm	0.53±0.09	0.48±0.09	0.44±0.08***	0.63±0.06***	0.57±0.13*
LVPWS, mm	0.71±0.14	0.58±0.08**	0.56±0.10***	0.83±0.15**	0.79±0.13*
LVAWD, mm	0.61±0.13	0.47±0.06***	0.49±0.08***	0.75±0.09***	0.68±0.10*
LVAWS, mm	0.77±0.13	0.61±0.08***	0.61±0.09***	0.94±0.14***	0.89±0.15***
LVEF, %	57.53±10.73	50.95±7.79*	50.06±8.82***	67.13±12.54**	65.84±7.79***
LVFS, %	29.98±7.03	25.56±4.90*	25.11±5.42***	36.85±9.69**	35.34±5.89**

LVEDD: left ventricle (LV) end-diastole diameter; LVESD: LV end-systole diameter; LVPWD: LV posterior wall at end-diastole; LVPWS: LV posterior wall at end-systole;

LVAWD: LV anterior wall at end-diastole; LVAWS: LV anterior wall at end-systole; LVEF: LV ejection fraction; LVFS: LV fractional shortening. **P*<0.05, ***P*<0.01, ****P*<0.001 *versus* NTG mice.

Table S2. Echocardiographic characteristics of mice at 3 months of age

Group	NTG	knockdown		overexpression	
		founder 24	founder 31	founder 2	founder 3
Number	n=55	n=7	n=33	n=13	n=41
LVEDD, mm	3.95±0.47	4.20±0.27	4.24±0.21**	3.56±0.48**	3.47±0.43***
LVESD, mm	3.01±0.54	3.21±0.37	3.31±0.37**	2.44±0.47***	2.37±0.48***
LVPWD, mm	0.57±0.12	0.45±0.10*	0.46±0.08***	0.66±0.09*	0.64±0.11**
LVPWS, mm	0.69±0.16	0.56±0.06*	0.53±0.09***	0.88±0.14***	0.86±0.16***
LVAWD, mm	0.62±0.13	0.44±0.09***	0.50±0.07***	0.82±0.15***	0.74±0.11***
LVAWS, mm	0.77±0.16	0.60±0.11**	0.60±0.08***	1.02±0.12***	0.96±0.16***
LVEF, %	48.35±10.93	47.43±8.90	44.57±8.16	60.44±9.70***	60.50±11.59***
LVFS, %	24.32±6.60	23.74±5.29	22.05±4.74	31.87±6.28***	32.09±7.77***

LVEDD: left ventricle (LV) end-diastole diameter; LVESD: LV end-systole diameter; LVPWD: LV posterior wall at end-diastole; LVPWS: LV posterior wall at end-systole;

LVAWD: LV anterior wall at end-diastole; LVAWS: LV anterior wall at end-systole; LVEF: LV ejection fraction; LVFS: LV fractional shortening. **P*<0.05, ***P*<0.01, ****P*<0.001 *versus* NTG mice.

Table S3. Echocardiographic characteristics of mice at 5 months of age

Group	NTG	knockdown		overexpression	
		founder 24	founder 31	founder 2	founder 3
Number	n=36	n=7	n=25	n=18	n=38
LVEDD, mm	4.02±0.50	4.26±0.20	4.51±0.37***	3.77±0.38	3.63±0.36***
LVESD, mm	3.02±0.64	3.42±0.25	3.67±0.29***	2.78±0.48	2.57±0.37***
LVPWD, mm	0.57±0.12	0.52±0.08	0.44±0.08***	0.61±0.09	0.63±0.11*
LVPWS, mm	0.69±0.17	0.52±0.07*	0.47±0.08***	0.78±0.12	0.82±0.12***
LVAWD, mm	0.64±0.12	0.55±0.05	0.49±0.06***	0.69±0.10	0.73±0.10***
LVAWS, mm	0.81±0.18	0.70±0.14	0.60±0.07***	0.88±0.12	0.97±0.16***
LVEF, %	49.94±13.74	41.22±4.76	37.91±8.54***	51.92±12.04	56.78±8.74*
LVFS, %	25.60±8.63	20.01±2.66	18.38±4.66***	26.52±7.43	29.40±5.74*

LVEDD: left ventricle (LV) end-diastole diameter; LVESD: LV end-systole diameter; LVPWD: LV posterior wall at end-diastole; LVPWS: LV posterior wall at end-systole;

LVAWD: LV anterior wall at end-diastole; LVAWS: LV anterior wall at end-systole; LVEF: LV ejection fraction; LVFS: LV fractional shortening. *P<0.05, **P<0.01, ***P<0.001 *versus* NTG mice.

Table S4. Echocardiographic characteristics of mice at 7 months of age

Group	NTG	knockdown		overexpression	
		Founder 24	Founder 31	Founder 2	Founder 3
Number	n=27	n=4	n=15	n=19	n=40
LVEDD, mm	4.14±0.45	4.47±0.33	4.66±0.37***	4.03±0.47	3.80±0.49**
LVESD, mm	3.07±0.55	3.41±0.41	3.75±0.41***	3.09±0.46	2.84±0.57
LVPWD, mm	0.60±0.11	0.49±0.04*	0.48±0.06***	0.57±0.13	0.57±0.13
LVPWS, mm	0.74±0.16	0.55±0.03*	0.53±0.07***	0.69±0.13	0.69±0.16
LVAWD, mm	0.67±0.14	0.53±0.03	0.54±0.05**	0.62±0.13	0.63±0.10
LVAWS, mm	0.89±0.16	0.66±0.08*	0.67±0.10***	0.80±0.19	0.80±0.17*
LVEF, %	51.31±10.70	47.52±7.22	40.23±6.39***	47.34±7.96	50.56±11.40
LVFS, %	26.24±6.63	23.82±4.14	19.64±3.59***	23.57±4.77	26.65±7.10

LVEDD: left ventricle (LV) end-diastole diameter; LVESD: LV end-systole diameter; LVPWD: LV posterior wall at end-diastole; LVPWS: LV posterior wall at end-systole;

LVAWD: LV anterior wall at end-diastole; LVAWS: LV anterior wall at end-systole; LVEF: LV ejection fraction; LVFS: LV fractional shortening. **P*<0.05, ***P*<0.01, ****P*<0.001 *versus* NTG mice.

Myocardium-specific Tomoregulin-1 knockdown (referred to as knockdown) and myocardium-specific Tomoregulin-1 overexpression mice (referred to as overexpression) as well as non-transgenic (NTG) littermates at 8 to 10 weeks of age were used for thoracic aorta constriction (TAC) operation. Briefly, mice were anesthetized by intraperitoneal injection 18ml/kg body weight tribromoethanol. The surgery was performed under passive respiration using a ventilator (125~150 times/min). The aorta was constricted between the origin of the right innominate and left common carotid arteries with a 6/0 polypropylene ligature using a blunted 27-gauge (0.41- mm OD) needle as a calibrator. The sham procedure was identical except that the aorta was not ligated. All the surviving mice were chosen for follow-up M-mode echocardiography at 4 weeks after surgery. The data were shown in the followed Supplemental Table S5-S6.

Table S5. Echocardiographic characteristics of mice at 4 weeks after Sham and TAC operation

	Sham			TAC		
Group	NTG	knockdown(24)	overexpression(2)	NTG	knockdown(24)	overexpression(2)
Number	n=55	n=7	n=13	n=28	n=8	n=17
LVEDD, mm	3.95±0.47	4.20±0.27	3.56±0.48**	3.89±0.57	4.26±0.26	3.86±0.30
LVESD, mm	3.01±0.54	3.21±0.37	2.44±0.47***	2.76±0.66	3.23±0.30	2.70±0.49
LVPWD, mm	0.57±0.12	0.45±0.10*	0.66±0.09*	0.77±0.15	0.69±0.10	0.66±0.14†
LVPWS, mm	0.69±0.16	0.56±0.06*	0.88±0.14***	0.97±0.22	0.82±0.12	0.90±0.17
LVAWD, mm	0.62±0.13	0.44±0.09***	0.82±0.15***	0.87±0.17	0.77±0.09	0.78±0.10
LVAWS, mm	0.77±0.16	0.60±0.11**	1.02±0.12***	1.16±0.19	1.04±0.18	1.04±0.16†
LVEF, %	48.35±10.93	47.43±8.90	60.44±9.70***	56.51±12.26	48.06±7.72	57.59±13.14
LVFS, %	24.32±6.60	23.74±5.29	31.87±6.28***	29.70±8.28	24.09±4.42	30.46±8.65

LVEDD: left ventricle (LV) end-diastole diameter; LVESD: LV end-systole diameter; LVPWD: LV posterior wall at end-diastole; LVPWS: LV posterior wall at end-systole;

LVAWD: LV anterior wall at end-diastole; LVAWS: LV anterior wall at end-systole; LVEF: LV ejection fraction; LVFS: LV fractional shortening.

**P*<0.05, ***P*<0.01, ****P*<0.001versus NTG-Sham mice; † *P*<0.05 versus NTG-TAC mice.

Table S6. Echocardiographic characteristics of mice at 4 weeks after Sham and TAC operation

	Sham			TAC		
Group	NTG	knockdown(31)	overexpression(3)	NTG	knockdown(31)	overexpression(3)
Number	n=55	n=33	n=41	n=28	n=14	n=28
LVEDD, mm	3.95±0.47	4.24±0.21**	3.47±0.43***	3.89±0.57	4.18±0.31	3.95±0.38
LVESD, mm	3.01±0.54	3.31±0.37**	2.37±0.48***	2.76±0.66	3.16±0.37†	2.85±0.54
LVPWD, mm	0.57±0.12	0.46±0.08***	0.64±0.11**	0.77±0.15	0.69±0.10	0.68±0.15†
LVPWS, mm	0.69±0.16	0.53±0.09***	0.86±0.16***	0.97±0.22	0.80±0.11††	0.90±0.17
LVAWD, mm	0.62±0.13	0.50±0.07***	0.74±0.11***	0.87±0.17	0.75±0.08†	0.78±0.11†
LVAWS, mm	0.77±0.16	0.60±0.08***	0.96±0.16***	1.16±0.19	1.02±0.14†	1.03±0.15††
LVEF, %	48.35±10.93	44.57±8.16	60.50±11.59***	56.51±12.26	48.78±8.44†	54.70±12.62
LVFS, %	24.32±6.60	22.05±4.74	32.09±7.77***	29.70±8.28	24.53±4.95	28.54±8.17

LVEDD: left ventricle (LV) end-diastole diameter; LVESD: LV end-systole diameter; LVPWD: LV posterior wall at end-diastole; LVPWS: LV posterior wall at end-systole;

LVAWD: LV anterior wall at end-diastole; LVAWS: LV anterior wall at end-systole; LVEF: LV ejection fraction; LVFS: LV fractional shortening.

*P<0.05, **P<0.01, ***P<0.001 versus NTG-sham mice; † P<0.05, †† P<0.01 versus NTG-TAC mice.

To verify whether the Tomoregulin-1 overexpression could affect the cardiac geometry and function in thoracic aorta constriction (TAC)-induced cardiac hypertrophy myocardium for long time, M-mode echocardiography was performed at 16 weeks after TAC operation on Tomoregulin-1 overexpression mice (referred to as overexpression) as well as non-transgenic (NTG) littermates. The data were shown in the followed Supplemental Table S7.

Table S7. Echocardiographic characteristics of mice at 16 weeks after TAC operation

	TAC	
Group	NTG	overexpression
Number	n=5	n=9
LVEDD, mm	4.83±0.85	3.77±0.69*
LVESD, mm	3.91±1.30	2.62±0.63*
LVPWD, mm	0.73±0.13	0.62±0.11
LVPWS, mm	0.87±0.12	0.88±0.10
LVAWD, mm	0.82±0.06	0.64±0.20
LVAWS, mm	0.87±0.17	0.94±0.15
LVEF, %	40.71±23.59	59.19±8.09*
LVFS, %	20.80±13.68	31.02±5.36*

LVEDD: left ventricle (LV) end-diastole diameter; LVESD: LV end-systole diameter; LVPWD: LV posterior wall at end-diastole; LVPWS: LV posterior wall at end-systole;

LVAWD: LV anterior wall at end-diastole; LVAWS: LV anterior wall at end-systole; LVEF: LV ejection fraction; LVFS: LV fractional shortening.

**P*<0.05 *versus* NTG mice.

Supplemental Figures

To verify whether the Tomoregulin-1 could affect the cardiac geometry and function, we generated two lines of myocardium-specific Tomoregulin-1 knockdown mice (founder 24 and 31) and Tomoregulin-1 overexpression mice (founder 2 and 3). M-mode echocardiography was performed at 1, 3, 5 and 7 months of age on each line of Tomoregulin-1 knockdown (referred to as knockdown) and overexpression mice (referred to as overexpression) as well as non-transgenic (NTG) littermates with the small animal echocardiography analysis system (Vevo770, Canada). The two lines of knockdown mice showed similar phenotypes of thin-walled ventricles and larger left ventricular diameters on M-mode echocardiography, and the two lines of overexpression mice showed similar phenotypes of thick-walled ventricles and smaller left ventricular. The M-mode echocardiography figures at 1, 3 and 7 months of age were showed in Figure S1.

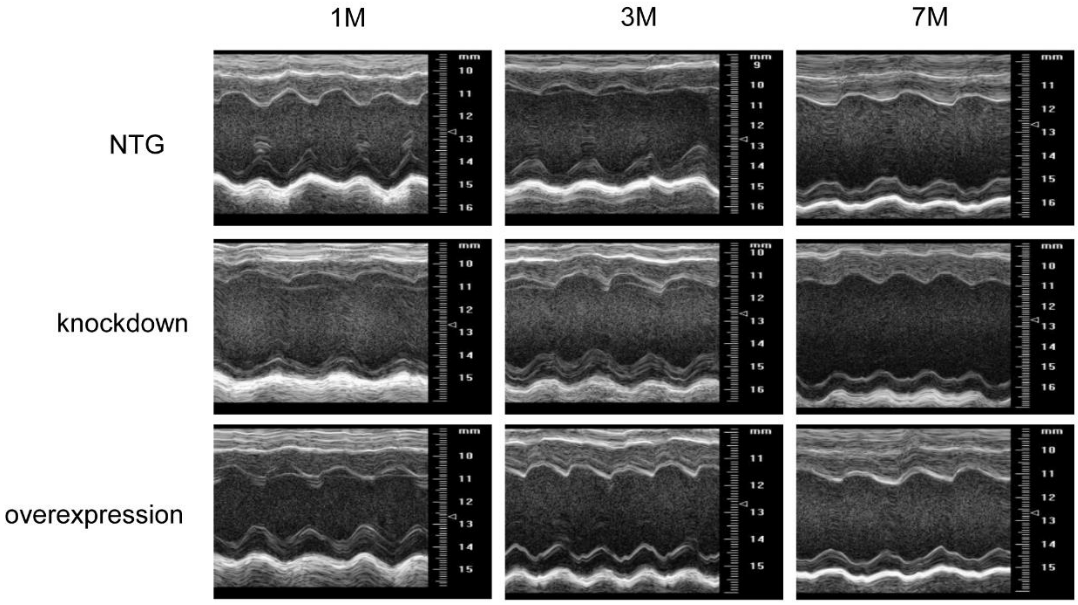

Figure S1. The M-mode echocardiography figures of NTG, Tomoregulin-1 knockdown and Tomoregulin-1 overexpression mice at 1, 3 and 7 months of age.

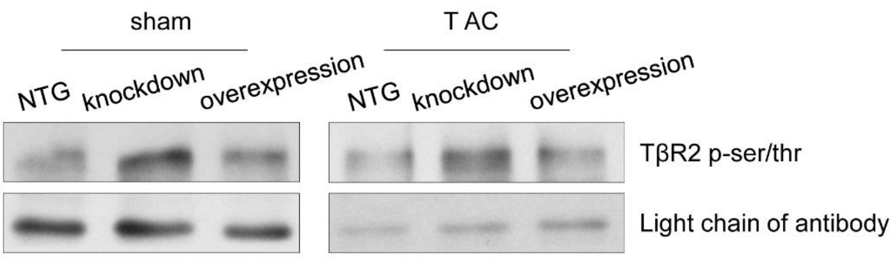

Figure S2. The phosphorylation level of TβR2 among Tomoregulin-1 knockdown mice, Tomoregulin-1 overexpression mice and their NTG littermates at 4 weeks after sham and TAC operation. The Western blot analysis of the level of phosphorylated TβR2 use light chain of antibody for normalization (*n* = 3 independent experiment).

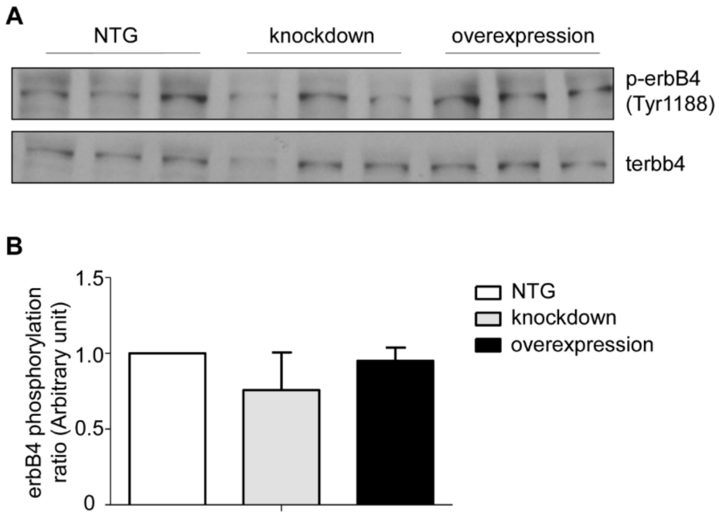

Figure S3. The phosphorylation level of erbB-4 were comparable among Tomoregulin-1 knockdown mice, Tomoregulin-1 overexpression mice and their NTG littermates. (A) The phosphorylation level of erbB-4 was measured by Western blot in the myocardium of NTG, Tomoregulin-1 knockdown and Tomoregulin-1 overexpression mice at 3 months of age. (B) The quantitative analysis of the level of phosphorylated erbB-4 use erbB-4 for normalization (n = 3 independent experiment).

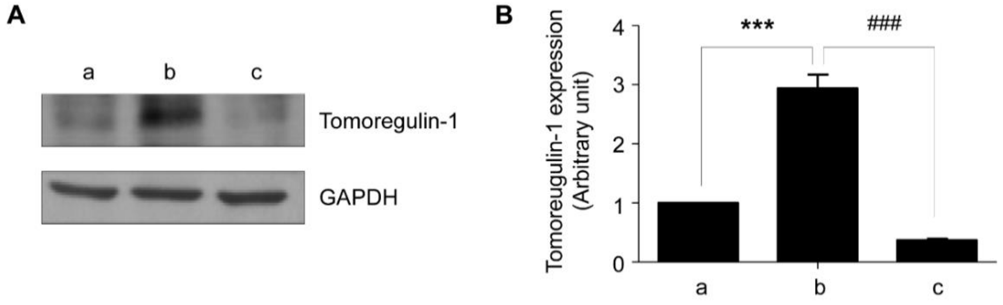

Figure S4. Two siRNA with high efficiency and showed synergic effects on the knockdown of Tomoregulin-1 in 293T cell. (A) The expression of Tomoregulin-1 in control 293T cell (a), co-transfection of the negative siRNA and the mouse Tomoregulin-1cDNA in 293T cell (b) and co-transfection of the two effective siRNA and the mouse Tomoregulin-1 cDNA in 293T cell (c). (B) The quantitative analysis of the expression of Tomoregulin-1 use GAPDH as normalization (n = 3 independent experiment, ***P<0.001 *versus* control 293T cell, ### P<0.001 *versus* co-transfection of the negative siRNA and the mouse Tomoregulin-1 cDNA in 293T cell).
